# Primary Hyperparathyroidism: An Overview

**DOI:** 10.1155/2011/251410

**Published:** 2011-06-02

**Authors:** Jessica MacKenzie-Feder, Sandra Sirrs, Donald Anderson, Jibran Sharif, Aneal Khan

**Affiliations:** ^1^Division of Endocrinology and Metabolism, Department of Medicine, University of British Columbia, Vancouver, BC, Canada V5Z 1M9; ^2^Division of Otolaryngology, Department of Surgery, University of British Columbia, Vancouver, BC, Canada V5Z 1M9; ^3^Department of Family Medicine, University of Saskatchwan, Regina, SK, Canada S4P 0W5; ^4^Alberta Children's Hospital, University of Calgary, 2888 Shaganappi Trail NW, Calgary, AB, Canada T3B 6A8

## Abstract

Primary hyperparathyroidism is a common condition that affects 0.3% of the general population. Primary and tertiary care specialists can encounter patients with primary hyperparathyroidism, and prompt recognition and treatment can greatly reduce morbidity and mortality from this disease. In this paper we will review the basic physiology of calcium homeostasis and then consider genetic associations as well as common etiologies and presentations of primary hyperparathyroidism. We will consider emerging trends in detection and measurement of parathyroid hormone as well as available imaging modalities for the parathyroid glands. Surgical indications and approach will be reviewed as well as medical management of primary hyperparathyroidism with bisphosphonates and calcimimetics.

## 1. Introduction

Parathyroid hormone is the chief regulator of calcium homeostasis in the human body. Primary hyperparathyroidism (PHPT) results from inappropriate overproduction of parathyroid hormone from one or many parathyroid gland(s) and presents with hypercalcemia. It is the third most common endocrine disorder affecting 0.3% of the general population, 1%–3% of postmenopausal women and a total population incidence of 21.6 cases per 100,000 person-years [1–3]. PHPT usually occurs as the result of sporadic parathyroid adenomas or carcinomas but can also be seen in association with multiple endocrine neoplasias and in rare genetic syndromes and metabolic diseases [4]. 

In children, primary hyperparathyroidism is rare. The most common cause is parathyroid adenoma, usually due to single gland disease, but severe neonatal hyperparathyroidism can also occur due to biallelic mutations in the calcium sensing receptor gene (*CASR*) with hypocalciuric hypercalcemia. 

It is important to differentiate primary from secondary and tertiary hyperparathyroidism. Secondary hyperparathyroidism occurs as a normal response to hypocalcemia due to diseases affecting the kidney (such as renal tubular acidosis), liver, intestines, and vitamin D deficiency. In newborn infants, maternal hypoparathyroidism with hypocalcemia, maternal pseudohypoparathyroidism, and rare genetic and metabolic syndromes can lead to secondary hyperparathyroidism. Tertiary hyperparathyroidism occurs in patients with long-standing secondary hyperparathyroidism who develop autonomous PTH production with hypercalcemia. The most common situation resulting in tertiary hyperparathyroidism is the patient with secondary hyperparathyroidism with renal failure who then receives a renal allograft [5–13]. This paper will focus on primary hyperparathyroidism.

## 2. Physiology of Calcium Regulation

Precise regulation of extracellular and intracellular calcium is essential for normal physiological processes such as cell signaling, neural function, muscular function (including cardiac contractility), hormone release and regulation, and bone metabolism [14]. Parathyroid hormone increases receptor-mediated tubular reabsorption of calcium in the kidney, stimulates release of skeletal calcium stores, upregulates 1-*α*-hydroxylase leading to increased 1,25-dihydroxy-vitamin D production and increased calcium reabsorption from the gastrointestinal tract [14].

Central to calcium regulation is the calcium-sensing receptor (CaSR) found primarily in the chief cells of the parathyroid glands [15]. The CaSR responds to the level of ionized calcium in the extracellular space and can upregulate or downregulate the secretion of parathyroid hormone. Newer evidence suggests that the CaSR plays a role independently of parathyroid hormone in the renal tubules to promote calcium secretion in the face of hypercalcemia [16].

## 3. Diagnosis of Primary Hyperparathyroidism

Primary hyperparathyroidism is diagnosed when PTH is elevated, in the context of hypercalcemia, in a patient with no history of renal disease. This is usually a result of inappropriate parathyroid hormone secretion from one or more of the parathyroid glands. Biochemical measurement of “intact” or “total” PTH is performed through immunoradiometric (IRMA) and immunochemiluminescent assays [17]. These “second-generation” assays have traditionally measured both the 1–84 amino acid sequence of PTH (considered the biologically active fragment) and other large fragments (with uncertain biological activity), such as the truncated 7–84 amino acid sequence PTH which can accumulate in patients with renal insufficiency [18, 19]. Measurement of PTH [1–84], widely called the “bioactive” or “3rd-generation assays” are considered the most precise method for measuring biologically active PTH using methods such as a two-site IRMA, a chemiluminescent enzymatic assay, or enzyme-linked immunosorbent assay (ELISA) [20–23].

## 4. Differential Diagnosis of Elevated PTH

Parathyroid hormone elevation can occur due to causes other than PHPT in patients with normal blood calcium levels. The most common cause is chronic kidney disease, but other causes include vitamin D deficiency, medications (such as lithium and thiazide diuretics), and familial hypocalciuric hypercalcemia (FHH) due to a heterozygous mutations of the calcium sensing receptor (*CASR*) gene [24]. The latter can be reasonably differentiated from PHPT based on the calcium to creatinine clearance ratio of less than 0.01 (mmol : mmol) with 85% sensitivity and 88% specificity [14, 25]. Vitamin D levels should be obtained in all patients with increased PTH levels and normal blood calcium levels since vitamin D deficiency can result in calcium levels which are lower than expected in patients with primary hyperparathyroidism [26].

## 5. Etiology/Epidemiology of Primary Hyperparathyroidism

Most patients with PHPT have a single adenoma (~80% of cases), but multigland disease can occur in 10%–15% of cases and double adenomas in 4%-5% [27]. Parathyroid carcinoma is a rare cause (usually less than 1% of patients) of hyperparathyroidism [28, 29].

Most cases of primary hyperparathyroidism are sporadic, but there is a higher incidence in patients with a history of neck irradiation [30], and approximately 5% of cases are familial as discussed below [27].

## 6. Genetic Causes of Primary Hyperparathyroidism

Mutations in a number of genes are responsible for familial adenomas or carcinomas presenting with hyperparathyroidism, but overall, these represent a minority of cases of PHPT. 

Germline mutations leading to loss of heterozygosity in the tumour suppressor genes *MEN1 *(menin) and *CDC73 *(formerly *HRPT2*) combined with a second mutation in somatic cells can increase the predisposition to parathyroid tumours [31, 32]. Mutations in only somatic cells in these genes have also been found in sporadic adenomas and carcinomas, respectively [33]. *MEN1 *is inherited in an autosomal dominant manner and is the most common cause of familial parathyroid adenomas and a rare cause of familial carcinomas [34]. Germline mutations in the *CDC73 *gene lead to autosomal dominant familial hyperparathyroidism tumour-jaw syndrome (HPT-JT), parathyroid carcinomas, and, in some cases, familial isolated hyperparathyroidism [32, 35–37]. HPT-JT is an autosomal dominant syndrome of primary hyperparathyroidism with parathyroid adenomas (with or without carcinomas), mandibular or maxillary fibro-osseous tumors, and renal tumours.

Mutations in the *RET *proto-oncogene, causing a number of different endocrine tumour syndromes, can be seen in MEN2A (associated with parathyroid adenomas) [38–40]. There are a number of rare cases of MEN4 due to *CDKN1B *mutations [41]. Two isolated consanguineous families, one with nephropathy, deafness, and hyperparathyroidism and another with hypophosphatemic rickets and hyperparathyroidism, have been reported but mutations were not described in either of the families [42, 43].

Mutations in the parathyroid hormone receptor *PTH1R *can be seen in skeletal dysplasias such as Murk Jansen metaphyseal chondrodysplasis [44], Blomstrand chondrodysplasia [45], Eiken skeletal dysplasias [46] and primary failure of tooth eruption [47] but thus far are not linked to parathyroid adenomas or carcinomas [48]. 

## 7. Clinical Presentation

The availability of the automated serum screening panel has changed the clinical presentation of PHPT. Whereas prior to the 1970s, this was a disease of recurrent kidney stones, osteitis fibrosa cystica, neuromuscular dysfunction characterized by type II muscle cell atrophy [49] and symptomatic hypercalcemia, it has now become an asymptomatic or mildly symptomatic disease detected by the incidental finding of hypercalcemia [50]. Of the classical symptoms, nephrolithiasis is the most common and occurs in 15%–20% of newly diagnosed patients with primary hyperparathyroidism [51].

PHPT affects compact bone more than trabecular bone with particular sensitivity in the cortices of long bones leading to subperiosteal bone resorption (seen as periosteal elevation on plain radiography) [52]. Although the lumbar spine is relatively spared in milder forms, some studies have shown vertebral osteopenia in 15% of patients at diagnosis which improves with parathyroidectomy [20, 51]. In advanced PHPT, the entire skeleton can be involved [51]. It is still unclear if PHPT imposes an independent fracture risk. A review of a large cohort of patients from the Mayo clinic suggested an overall increased fracture risk at all sites except the hip [53]. However, the cortical bone thinning effects may be counterbalanced by increased bone diameter and PTH-mediated periosteal apposition and the preservation of bone microarchitecture [54]. 

Less specific features of primary hyperparathyroidism include fatigue, muscle weakness, mild cognitive disturbances, hypertension, left ventricular hypertrophy, valvular calcification, and cardiovascular mortality [20, 27], but little data exists on whether these entities are treatable using standard approaches to manage primary hyperparathyroidism.

## 8. Management of Primary Hyperparathyroidism

### 8.1. Surgical Management

The only cure for primary hyperparathyroidism due to parathyroid adenomas is surgical resection of the culprit gland or glands. In 2008, The Third International Workshop on Asymptomatic Primary Hyperparathyroidism revised the indications for surgery in asymptomatic patients—these include age less than 50 years, serum calcium 0.25 mmol/L above the upper limit of normal, creatinine clearance <60 mL/min, DXA t-score <-2.5 at any site, and/or previous fragility fracture [58]. An isolated elevation of 24 hour urine calcium is no longer an indication for surgery although this measurement may be necessary to rule out FHH which, although rare, is most commonly the result of a heterozygous mutation of the *CASR* gene. This is very different from neonatal severe hyperparathyroidism, due to biallelic mutations in the *CASR* gene, in which the hypercalcemia is fatal unless recognized early and subtotal parathyroidectomy is undertaken upon diagnosis [59]. Surgical treatment may also be the preferred option to manage pregnant women, children, and adolescents with hyperparathyroidism [6, 60]. Parathyroid carcinomas present with high calcium levels and require *en bloc* resection of an enlarged gland adherent to adjacent tissue [61]. 

Bilateral neck exploration with direct visualization and identification of all abnormal parathyroid glands with subsequent removal was previously considered the gold standard of care. Experienced surgeons reported to be able to identify affected glands in 95% of cases [14]. Unilateral neck exploration is also highly effective, because PTH has a short half-life of less than five minutes and can be measured after and before adenoma resection to ensure that the culprit gland has been removed. If levels remain high after resection of the suspected gland, unilateral neck exploration can be converted to bilateral neck exploration for direct visualization and identification of missed hypersecreting parathyroid tissue. 

The new standard for most surgeons is preoperative radiologic localization of adenomas to direct a focused parathyroidectomy using unilateral neck exploration and adjunctive intraoperative PTH [65–67]. Intraoperative hand held gamma probe identification of *in situ* and resected radiolabelled hyperplastic parathyroid glands can be helpful.

In contrast to sporadic primary hyperparathyroidism, familial isolated primary hyperparathyroidism (FIHPT) is characterized by earlier onset disease, higher incidence of multiglandular involvement and a higher recurrence rate. Subtotal parathyroidectomy or total excision with autotransplantation is recommended for multi-glandular disease. There is a risk of permanent hypocalcemia. It is reported that many patients with FIHPT return to normocalcemia after excision of single-gland disease [85]. The use of preoperative localization and an intraoperative parathyroid hormone assay permits limited resection of only hypersecreting glands. Limited parathyroidectomy allows successful single-gland excision in many patients with FIHPT, thus decreasing the risk of hypoparathyroidism. 

Familial hyperparathyroidism associated with MEN1 and MEN2A and familial isolated hyperparathyroidism are also managed surgically [62–64]. MEN1-associated hyperparathyroidism patients present earlier than sporadic patients but with similar symptoms. There is an asymmetrical multi-glandular involvement with a greater size difference between glands than in the hyperplasia of sporadic primary hyperparathyroidism [86]. The multiple monoclonal tumors and polyclonal hyperplasias which develop asynchronously are surgically managed. At exploration, all glands should be visualized and those normal glands to remain *in situ* should be marked for later localization. If one or two normal glands are found, then only the hyperplastic glands should be removed. If all glands are hyperplastic, total parathyroidectomy and immediate autotransplantation will obviate the need to subsequently explore a neck scarred from previous surgery. Generally, the thymus is also removed because of the possible presence of diseased supernumerary glands within it. Because unrecognized MEN1 patients should not be explored, a diagnosis should be obtained before surgical exploration in patients with hyperparathyroidism [87, 88].

A minority of MEN2 patients develop hyperparathyroidism which, when present, is characteristically mild. At total thyroidectomy for medullary carcinoma, only hypertrophied parathyroid glands should be removed after all are identified and marked [89].

Complications from surgery for parathyroidectomy include persistent hyperparathyroidism if insufficient disease-causing tissue is removed (which can increase the risk of fractures), recurrent laryngeal nerve injury, hematoma, infection, pneumonia, transient or permanent postoperative hypocalcemia, and seizures from hypocalcemia and hypomagnesemia [68–71]. Preoperative elevation of serum alkaline phosphatase may signal the possibility of future postoperative bone hunger and depressed postoperative serum calcium levels. Minimally invasive techniques and an experienced surgeon can reduce the risk of complications [68, 72]. 

Hyperparathyroidism is a condition that disproportionately affects older patients who have traditionally been considered a population at higher operative risk, but several recent studies have not shown this assumption to be justified [73, 74]. Parathyroidectomy should, therefore, be considered first-line therapy even for the frail elderly with true symptoms of hyperparathyroidism. This must be a joint decision between the patient, the treating physician, and the surgeon.

### 8.2. Identification of Suspect Hypersecreting Parathyroid Glands

If a surgeon is planning an open parathyroidectomy, where all 4 glands are directly visualized, no preoperative imaging may be required and the use of preoperative imaging in such circumstances is a matter of personal preference for the surgeon. However, preoperative imaging is needed if the surgeon is planning minimally invasive parathyroidectomy, where only one side of the neck is exposed. Preoperative imaging can also be helpful in patients with previous neck surgery in whom scar tissue can make direct visualization more challenging. Of the available imaging techniques, the most successful modality is the ^99^technetium-labelled sestamibi-single photon emission CT identifying up to 89% of single parathyroid adenomas [55, 56, 90, 91]. ^99^Technetium-labelled sestamibi (^99^mTc MIBI) is taken up by parathyroid and thyroid tissue. Uptake is enhanced and prolonged in adenomatous and hyperplastic parathyroids [91]. Ultrasound is the second most useful modality and when used with ^99^mTc MIBI preoperatively can enhance adenoma detection rates. Plain computed tomography and magnetic resonance imaging are less useful except in the context of persistent or ectopic production [57].

### 8.3. Revision Surgery

About 5% of patients undergoing parathyroidectomy will exhibit persistent hypercalcemia because of insufficient removal of disease-causing tissue. The localization of persistent disease can be anticipated based upon verification of the diagnosis, reviewing pathology slides and operative reports, interviewing previous surgeons and by adding localization procedures to those previously mentioned [92]. Gadolinium-enhanced magnetic resonance MRI, CT with intravenous contrast, angiography, and selective venous sampling for PTH can aid with localization. Most missed glands are found in the neck. Some surgeons enter the operative site lateral to the strap muscles but medial to the sternocleidomastoid muscle and great neck vessels to avoid a scarred midline field [93]. Upper mediastinal locations may be approached via an upper-third median sternotomy or by video-assisted thoracic surgery (VATS). If repeat operation is unsuccessful, observation and pulsed localization testing may allow detection as hyperfunctioning parathyroid tissue enlarges. 

### 8.4. Medical Management

Not all cases of primary hyperthyroidism require surgical management. For primary hyperparathyroidism due to parathyroid adenomas, surgery has a very high long-term success rate and minimal morbidity. In contrast, targeted medical therapy with calcimimetics, such as cinacalcet, is very costly, and both cinacalcet and bisphosphonates can cause unpleasant side effects. When the cause of hyperparathyroidism is not primary pathology in the parathyroid glands, nonsurgical management may be indicated, and treatment should be aimed at treating the underlying cause—this principle will be relevant to some of the neonatal causes and rare syndromes of hyperparathyroidism.

In some situations, however, medical management is a consideration for patients with PHPT who are asymptomatic from their disease or for patients who are not candidates for surgery. The cornerstones of medical management include bone protection with the use of bisphosphonates and lowering of calcium level with calcimimetics.

Multiple studies have shown that bisphosphonates improve bone mineral density on DXA scan in patients with primary hyperparathyroidism. Alendronate has been the most well-studied bisphosphonate and has shown increases in bone mineral density of the lumbar spine and the proximal femur when compared to placebo [75–77]. When using bisphosphonates, the risk of potential complications, among them osteonecrosis of the jaw, should be considered [55, 78].

Hormone replacement therapy has also been studied in postmenopausal women with PHPT and it was found to have similar bone protective effects as in normocalcemic postmenopausal women without PHPT [79]. This should only be used as therapy for PHPT in women who are already taking HRT for perimenopausal symptoms.

Calcimimetics, like cinacalcet, are designed to allosterically modify the calcium-sensing receptor, thus sensitizing it to circulating calcium levels and downregulating PTH transcription, secretion, and parathyroid cell proliferation [80, 81]. Studies show that cinacalcet (versus placebo) effectively lowers both calcium levels and PTH levels, thus normalizing the abnormal biochemistry associated with PHPT [82]. There is no significant increase in bone mineral density for patients taking cinacalcet versus placebo as was seen in the trials with bisphosphonates. However, there was no significant loss of bone mineral density in either the placebo or the cinacalcet groups [83]. Specific indications for use of cinacalcet are limited to situations in which symptomatic hypercalcemia needs to be controlled in a patient who cannot undergo surgery. It can sometimes also be used as a trial to see if lowering calcium improves symptoms in someone who is considering parathyroidectomy. If preservation of bone mineral density is the goal of treating asymptomatic PHPT, then the agent of choice is a bisphosphonate [50].

## 9. Conclusion

Primary hyperparathyroidism is one of the most common endocrinological disorders. In rare circumstances, primary hyperparathyroidism is associated with several familial syndromes. Primary hyperparathyroidism is most frequently identified incidentally on automated multichannel blood screening panels and is very often asymptomatic at the time of diagnosis. Parathyroidectomy remains the definitive cure for the disease, and some controversy surrounds the optimal surgical technique for the procedure. Medical therapy with the goal of bone preservation is an option for patients without symptoms, and calcimimetics effectively normalize calcium levels but have specific indications for use. Medical therapy may gain popularity for patients with hypercalcemia who cannot undergo surgery.

## Abbreviations

CASR: Calcium-sensing receptor
CT: Computed tomography
DXA: Dual X-ray absorptiometry
ELISA: Enzyme-linked immunosorbent assay
FHH: Familial hypocalciuric hypercalcemia
FIHPT: Familial isolated primary hyperparathyroidism
HPT: Hyperparathyroidism
MRI: Magnetic resonance imaging
PHPT: Primary hyperparathyroidism
PTH: Parathyroid hormone
SPECT/MIBI: Sestamibi-single photon emission computed tomography
VATS: Video-assisted thoracic surgery.

## References

[1] Wermers RA, Khosla S, Atkinson EJ (2006). Incidence of primary hyperparathyroidism in Rochester, Minnesota, 1993–2001: an update on the changing epidemiology of the disease. *Journal of Bone and Mineral Research*.

[2] Mihai R, Wass JA, Sadler GP (2008). Asymptomatic hyperparathyroidism—need for multicentre studies. *Clinical Endocrinology*.

[3] Melton LJ (2002). The epidemiology of primary hyperparathyroidism in North America. *Journal of Bone and Mineral Research*.

[4] Unger S, Paul DA, Nino MC (2005). Mucolipidosis II presenting as severe neonatal hyperparathyroidism. *European Journal of Pediatrics*.

[5] George J, Acharya SV, Bandgar TR, Menon PS, Shah NS (2010). Primary hyperparathyroidism in children and adolescents. *Indian Journal of Pediatrics*.

[6] Durkin ET, Nichol PF, Lund DP, Chen H, Sippel RS (2010). What is the optimal treatment for children with primary hyperparathyroidism?. *Journal of Pediatric Surgery*.

[7] Igarashi T, Sekine Y, Kawato H, Kamoshita S, Saigusa Y (1992). Transient neonatal distal renal tubular acidosis with secondary hyperparathyroidism. *Pediatric Nephrology*.

[8] Glass EJ, Barr DG (1981). Transient neonatal hyperparathyroidism secondary to maternal pseudohypoparathyroidism. *Archives of Disease in Childhood*.

[9] Sathasivam A, Garibaldi L, Murphy R, Ibrahim J (2006). Transient neonatal hyperparathyroidism: a presenting feature of mucolipidosis type II. *Journal of Pediatric Endocrinology and Metabolism*.

[10] Minagawa M, Yasuda T, Kobayashi Y, Niimi H (1995). Transient pseudohypoparathyroidism of the neonate. *European Journal of Endocrinology*.

[11] Bai M, Pearce SH, Kifor O (1997). In vivo and in vitro characterization of neonatal hyperparathyroidism resulting from a de novo, heterozygous mutation in the Ca2+-sensing receptor gene: normal maternal calcium homeostasis as a cause of secondary hyperparathyroidism in familial benign hypocalciuric hypercalcemia. *Journal of Clinical Investigation*.

[12] Loughead JL, Mughal Z, Mimouni F, Tsang RC, Oestreich AE (1990). Spectrum and natural history of congenital hyperparathyroidism secondary to maternal hypocalcemia. *American Journal of Perinatology*.

[13] Pazzaglia UE, Beluffi G, Bianchi E, Castello A, Coci A, Marchi A (1989). Study of the bone pathology in early mucolipidosis II (I-cell disease). *European Journal of Pediatrics*.

[14] Fraser WD (2009). Hyperparathyroidism. *The Lancet*.

[15] Brown EM (2007). Clinical lessons from the calcium-sensing receptor. *Nature Clinical Practice Endocrinology and Metabolism*.

[16] Kantham L, Quinn SJ, Egbuna OI (2009). The calcium-sensing receptor (CaSR) defends against hypercalcemia independently of its regulation of parathyroid hormone secretion. *American Journal of Physiology—Endocrinology and Metabolism*.

[17] Endres DB, Villanueva R, Sharp CFJ, Singer FR (1991). Immunochemiluminometric and immunoradiometric determinations of intact and total immunoreactive parathyrin: performance in the differential diagnosis of hypercalcemia and hypoparathyroidism. *Clinical Chemistry*.

[18] Nussbaum SR, Zahradnik RJ, Lavigne JR (1987). Highly sensitive two-site immunoradiometric assay of parathyrin, and its clinical utility in evaluating patients with hypercalcemia. *Clinical Chemistry*.

[19] Gao P, Scheibel S, D’Amour P (2001). Development of a novel immunoradiometric assay exclusively for biologically active whole parathyroid hormone 1–84: implications for improvement of accurate assessment of parathyroid function. *Journal of Bone and Mineral Research*.

[20] Silverberg SJ, Gao P, Brown I, Logerfo P, Cantor TL, Bilezikian JP (2003). Clinical utility of an immunoradiometric assay for parathyroid hormone (1–84) in primary hyperparathyroidism. *Journal of Clinical Endocrinology and Metabolism*.

[21] Melamed ML, Eustace JA, Plantinga LC (2008). Third-generation parathyroid hormone assays and all-cause mortality in incident dialysis patients: the CHOICE study. *Nephrology Dialysis Transplantation*.

[22] Ljungdahl N, Haarhaus M, Linder C, Magnusson P (2006). Comparison of 3 third-generation assays for bio-intact parathyroid hormone. *Clinical Chemistry*.

[23] Kazama JJ, Yamamoto S, Kameda S (2005). Direct comparison between two 1–84PTH assays in dialysis patients. *Nephron Clinical Practice*.

[24] Pollak MR, Brown EM, Chou YH (1993). Mutations in the human Ca(2+)-sensing receptor gene cause familial hypocalciuric hypercalcemia and neonatal severe hyperparathyroidism. *Cell*.

[25] Fuleihan G-H (2002). Familial benign hypocalciuric hypercalcemia. *Journal of Bone and Mineral Research*.

[26] Eastell R, Arnold A, Brandi ML (2009). Diagnosis of asymptomatic primary hyperparathyroidism: proceedings of the third international workshop. *Journal of Clinical Endocrinology and Metabolism*.

[27] Felger EA, Kandil E (2010). Primary hyperparathyroidism. *Otolaryngologic Clinics of North America*.

[28] Marx SJ (2000). Hyperparathyroid and hypoparathyroid disorders. *New England Journal of Medicine*.

[29] Shane E (2001). Clinical review 122: parathyroid carcinoma. *Journal of Clinical Endocrinology and Metabolism*.

[30] Stephen AE, Chen KT, Milas M, Siperstein AE (2004). The coming of age of radiation-induced hyperparathyroidism: evolving patterns of thyroid and parathyroid disease after head and neck irradiation. *Surgery*.

[31] Teh BT, Kytola S, Farnebo F (1998). Mutation analysis of the MEN1 gene in multiple endocrine neoplasia type 1, familial acromegaly and familial isolated hyperparathyroidism. *Journal of Clinical Endocrinology and Metabolism*.

[32] Carpten JD, Robbins CM, Villablanca A (2002). HRPT2, encoding parafibromin, is mutated in hyperparathyroidism-jaw tumor syndrome. *Nature Genetics*.

[33] Tanaka C, Yoshimoto K, Yamada S (1998). Absence of germ-line mutations of the multiple endocrine neoplasia type 1 (MEN1) gene in familial pituitary adenoma in contrast to MEN1 in Japanese. *Journal of Clinical Endocrinology and Metabolism*.

[34] Thakker RV (1998). Multiple endocrine neoplasia—syndromes of the twentieth century. *Journal of Clinical Endocrinology and Metabolism*.

[35] Shattuck TM, Valimaki S, Obara T (2003). Somatic and germ-line mutations of the HRPT2 gene in sporadic parathyroid carcinoma. *New England Journal of Medicine*.

[36] Howell VM, Haven CJ, Kahnoski K (2003). HRPT2 mutations are associated with malignancy in sporadic parathyroid tumours. *Journal of Medical Genetics*.

[37] Simonds WF, Robbins CM, Agarwal SK, Hendy GN, Carpten JD, Marx SJ (2004). Familial isolated hyperparathyroidism is rarely caused by germline mutation in HRPT2, the gene for the hyperparathyroidism-jaw tumor syndrome. *Journal of Clinical Endocrinology and Metabolism*.

[38] Shirahama S, Ogura K, Takami H (1998). Mutational analysis of the RET proto-oncogene in 71 Japanese patients with medullary thyroid carcinoma. *Journal of Human Genetics*.

[39] Elisei R, Romei C, Cosci B (2007). RET genetic screening in patients with medullary thyroid cancer and their relatives: experience with 807 individuals at one center. *Journal of Clinical Endocrinology and Metabolism*.

[40] Eng C, Crossey PA, Mulligan LM (1995). Mutations in the RET proto-oncogene and the von Hippel-Lindau disease tumour suppressor gene in sporadic and syndromic phaeochromocytomas. *Journal of Medical Genetics*.

[41] Pellegata NS, Quintanilla-Martinez L, Siggelkow H (2006). Germ-line mutations in p27Kip1 cause a multiple endocrine neoplasia syndrome in rats and humans. *Proceedings of the National Academy of Sciences of the United States of America*.

[42] Edwards BD, Patton MA, Dilly SA, Eastwood JB (1989). A new syndrome of autosomal recessive nephropathy, deafness, and hyperparathyroidism. *Journal of Medical Genetics*.

[43] Brownstein CA, Adler F, Nelson-Williams C (2008). A translocation causing increased alpha-klotho level results in hypophosphatemic rickets and hyperparathyroidism. *Proceedings of the National Academy of Sciences of the United States of America*.

[44] Schipani E, Kruse K, Juppner H (1995). A constitutively active mutant PTH-PTHrP receptor in Jansen-type metaphyseal chondrodysplasia. *Science*.

[45] Jobert AS, Zhang P, Couvineau A (1998). Absence of functional receptors for parathyroid hormone and parathyroid hormone-related peptide in Blomstrand chondrodysplasia. *Journal of Clinical Investigation*.

[46] Eiken M, Prag J, Petersen KE, Kaufmann HJ (1984). A new familial skeletal dysplasia with severely retarded ossification and abnormal modeling of bones especially of the epiphyses, the hands, and feet. *European Journal of Pediatrics*.

[47] Decker E, Stellzig-Eisenhauer A, Fiebig BS (2008). PTHR1 loss-of-function mutations in familial, nonsyndromic primary failure of tooth eruption. *American Journal of Human Genetics*.

[48] http://www.ncbi.nlm.nih.gov/omim.

[49] Patten BM, Pages M (1984). Severe neurological disease associated with hyperparathyroidism. *Annals of Neurology*.

[50] Khan AA, Bilezikian JP, Potts JTJ (2009). The diagnosis and management of asymptomatic primary hyperparathyroidism revisited. *Journal of Clinical Endocrinology and Metabolism*.

[51] Bilezikian JP (2005). Anabolic therapy for osteoporosis. *International Journal of Fertility and Women’s Medicine*.

[52] Khan AHJ, Pender A, Wei X, Potter M (2008). I-cell disease (mucolipidosis II) presenting as neonatal fractures: a case for continued monitoring of serum parathyroid hormone levels. *Clinical Pediatric Endocrinology*.

[53] Khosla S, Melton L, Wermers RA, Crowson CS, O’Fallon W, Riggs B (1999). Primary hyperparathyroidism and the risk of fracture: a population-based study. *Journal of Bone and Mineral Research*.

[54] Macdonald HM, Nishiyama KK, Hanley DA, Boyd SK (2011). Changes in trabecular and cortical bone microarchitecture at peripheral sites associated with 18months of teriparatide therapy in postmenopausal women with osteoporosis. *Osteoporosis International*.

[55] Patel CN, Scarsbrook AF (2009). Multimodality imaging in hyperparathyroidism. *Postgraduate Medical Journal*.

[56] Gayed IW, Kim EE, Broussard WF (2005). The value of 99mTc-sestamibi SPECT/CT over conventional SPECT in the evaluation of parathyroid adenomas or hyperplasia. *Journal of Nuclear Medicine*.

[57] Johnson NA, Tublin ME, Ogilvie JB (2007). Parathyroid imaging: technique and role in the preoperative evaluation of primary hyperparathyroidism. *American Journal of Roentgenology*.

[58] Bilezikian JP, Khan AA, Potts JTJ (2009). Guidelines for the management of asymptomatic primary hyperparathyroidism: summary statement from the third international workshop. *Journal of Clinical Endocrinology and Metabolism*.

[59] Rhone DP (1975). Primary neonatal hyperparathyroidism. Report of a case and review of the literature. *American Journal of Clinical Pathology*.

[60] McMullen TP, Learoyd DL, Williams DC, Sywak MS, Sidhu SB, Delbridge LW (2010). Hyperparathyroidism in pregnancy: options for localization and surgical therapy. *World Journal of Surgery*.

[61] Fujimoto Y, Obara T, Ito Y, Kanazawa K, Aiyoshi Y, Nobori M (1984). Surgical treatment of ten cases of parathyroid carcinoma: importance of an initial en bloc tumor resection. *World Journal of Surgery*.

[62] Hellman P, Skogseid B, Oberg K, Juhlin C, Akerström G, Rastad J (1998). Primary and reoperative parathyroid operations in hyperparathyroidism of multiple endocrine neoplasia type 1. *Surgery*.

[63] Dralle H, Scheumann GF, Kotzerke J, Brabant EG (1992). Surgical management of MEN 2. *Recent Results in Cancer Research*.

[64] Kassem M, Kruse TA, Wong FK, Larsson C, Teh BT (2000). Familial isolated hyperparathyroidism as a variant of multiple endocrine neoplasia type 1 in a large Danish pedigree. *Journal of Clinical Endocrinology and Metabolism*.

[65] Carneiro-Pla DM, Solorzano CC, Irvin GL (2006). Consequences of targeted parathyroidectomy guided by localization studies without intraoperative parathyroid hormone monitoring. *Journal of the American College of Surgeons*.

[66] Sackett WR, Barraclough BH, Sidhu S, Reeve TS, Delbridge LW (2002). Minimal access thyroid surgery: is it feasible, is it appropriate?. *ANZ Journal of Surgery*.

[67] Baliski CR, Stewart JK, Anderson DW, Wiseman SM, Bugis SP (2005). Selective unilateral parathyroid exploration: an effective treatment for primary hyperparathyroidism. *American Journal of Surgery*.

[68] Richmond BK, Eads K, Flaherty S, Belcher M, Runyon D (2007). Complications of thyroidectomy and parathyroidectomy in the rural community hospital setting. *American Surgeon*.

[69] Norman J, Chheda H, Farrell C (1998). Minimally invasive parathyroidectomy for primary hyperparathyroidism: decreasing operative time and potential complications while improving cosmetic results. *American Surgeon*.

[70] Davies DR, Friedman M (1966). Complications after parathyroidectomy. Fractures from low calcium and magnesium convulsions. *Journal of Bone and Joint Surgery*.

[71] Anderberg B, Gillquist J, Larsson L, Lundstrom B (1981). Complications to subtotal parathyroidectomy. *Acta Chirurgica Scandinavica*.

[72] Jatzko GR, Lisborg PH, Muller MG, Wette VM (1994). Recurrent nerve palsy after thyroid operations—principal nerve identification and a literature review. *Surgery*.

[73] Shin SH, Holmes H, Bao R (2009). Outpatient minimally invasive parathyroidectomy is safe for elderly patients. *Journal of the American College of Surgeons*.

[74] Stechman MJ, Weisters M, Gleeson FV, Sadler GP, Mihai R (2009). Parathyroidectomy is safe and improves symptoms in elderly patients with primary hyperparathyroidism (PHPT). *Clinical Endocrinology*.

[75] Khan AA, Bilezikian JP, Kung A, Dubois SJ, Standish TI, Syed ZA (2009). Alendronate therapy in men with primary hyperparathyroidism. *Endocrine Practice*.

[76] Rossini M, Gatti D, Girardello S, Braga V, James G, Adami S (2000). Effects of two intermittent alendronate regimens in the prevention or treatment of postmenopausal osteoporosis. *Bone*.

[77] Parker CR, Blackwell PJ, Fairbairn KJ, Hosking DJ (2002). Alendronate in the treatment of primary hyperparathyroid-related osteoporosis: a 2-year study. *Journal of Clinical Endocrinology and Metabolism*.

[78] Otto S, Abu-Id MH, Fedele S (2011). Osteoporosis and bisphosphonates-related osteonecrosis of the jaw: not just a sporadic coincidence—a multi-centre study. *Journal of Cranio-Maxillofacial Surgery*.

[79] Grey AB, Stapleton JP, Evans MC, Tatnell MA, Reid IR (1996). Effect of hormone replacement therapy on bone mineral density in postmenopausal women with mild primary hyperparathyroidism: a rRandomized, controlled trial. *Annals of Internal Medicine*.

[80] Nemeth EF, Heaton WH, Miller M (2004). Pharmacodynamics of the type II calcimimetic compound cinacalcet HCl. *Journal of Pharmacology and Experimental Therapeutics*.

[81] Hofer AM, Brown EM (2003). Extracellular calcium sensing and signalling. *Nature Reviews Molecular Cell Biology*.

[82] Peacock M, Bilezikian JP, Klassen PS, Guo MD, Turner SA, Shoback D (2005). Cinacalcet hydrochloride maintains long-term normocalcemia in patients with primary hyperparathyroidism. *Journal of Clinical Endocrinology and Metabolism*.

[83] Shoback DM, Bilezikian JP, Turner SA, McCary LC, Guo MD, Peacock M (2003). The calcimimetic cinacalcet normalizes serum calcium in subjects with primary hyperparathyroidism. *Journal of Clinical Endocrinology and Metabolism*.

[84] Friedman E, Sakaguchi K, Bale AE (1989). Clonality of parathyroid tumors in familial multiple endocrine neoplasia type 1. *New England Journal of Medicine*.

[85] Carneiro DM, Irvin GLR, Inabnet WB (2002). Limited versus radical parathyroidectomy in familial isolated primary hyperparathyroidism. *Surgery*.

[86] Levine DS, Belzberg AS, Wiseman SM (2009). Hybrid SPECT/CT imaging for primary hyperparathyroidism: case reports and pictorial review. *Clinical Nuclear Medicine*.

[87] van Heerden JA, Grant CS (1991). Surgical treatment of primary hyperparathyroidism: an institutional perspective. *World Journal of Surgery*.

[88] Hellman P, Skogseid B, Juhlin C, Akerström G, Rastad J (1992). Findings and long-term results of parathyroid surgery in multiple endocrine neoplasia type 1. *World Journal of Surgery*.

[89] Snow KJ, Boyd AER (1994). Management of individual tumor syndromes: medullary thyroid carcinoma and hyperparathyroidism. *Endocrinology and Metabolism Clinics of North America*.

[90] Levine DS, Wiseman SM (2010). Fusion imaging for parathyroid localization in primary hyperparathyroidism. *Expert Review of Anticancer Therapy*.

[91] Swanson TW, Chan SK, Jones SJ (2010). Determinants of Tc-99m sestamibi SPECT scan sensitivity in primary hyperparathyroidism. *American Journal of Surgery*.

[92] Gaz RD, Randolf GW (2003). Revision parathyroid surgery. *Surgery of the Thyroid and Parathyroid Glands*.

[93] Cameron JL (2001). *Current Surgical Therapy*.

